# Distinct Features of Cerebral Blood Flow and Spontaneous Neural Activity as Integrated Predictors of Early Response to Antidepressants

**DOI:** 10.3389/fpsyt.2021.788398

**Published:** 2022-01-18

**Authors:** Zhenghua Hou, Tong Li, Xiaofu He, Yuqun Zhang, Huanxin Chen, Wenhao Jiang, Yingying Yin, Yonggui Yuan

**Affiliations:** ^1^Department of Psychosomatics and Psychiatry, Institute of Psychosomatics, School of Medicine, Affiliated Zhongda Hospital, Southeast University, Nanjing, China; ^2^Department of Psychiatry, The New York State Psychiatric Institute, Columbia University Medical Center, New York, NY, United States; ^3^Department of Information Engineering, Harbin Institute of Technology, Harbin, China; ^4^Key Laboratory of Cognition and Personality, Ministry of Education, School of Psychology, Southwest University, Chongqing, China

**Keywords:** predictors, early antidepressants response, support vector machine, cerebral blood flow, amplitude of low-frequency fluctuation

## Abstract

**Aims:**

The purpose of this study is to explore whether pre-treatment features of brain function can discriminate non-responders to antidepressant medication in the early phase.

**Methods:**

Forty-four treatment-responsive depressed (RD) patients, 36 non-responsive depressed (NRD) patients, and 42 healthy controls (HCs) were recruited. Regional cerebral blood flow (CBF) and amplitude of low-frequency fluctuation (ALFF) values were calculated for all subjects. Correlation analyses were used to explore the relationship between symptom improvement and CBF/ALFF. Receiver operating characteristics (ROC) and the 10-fold cross-validation support vector machine (SVM) classifier were applied for the discrimination of treatment response.

**Results:**

Compared with the HCs, the RD and NRD groups exhibited lower CBF and ALFF in the right posterior lobe of the cerebellum. Compared with the NRD group, the RD group showed distinct CBF patterns in the left frontal striatal regions and right frontal cerebellar regions, as well as distinct ALFF features in the left frontoparietal striatum and right frontotemporal striatal cerebellar regions. The ROC and SVM classifier revealed the optimal power to distinguish the RD and NRD groups based on the combined measures (i.e., CBF and ALFF).

**Conclusion:**

Distinct features of CBF and ALFF in the frontal striatal network may serve as promising neuroimaging predictors for identifying patients with blunted responsiveness, which may facilitate personalized antidepressant treatment.

## Introduction

Despite their importance for treating major depressive disorder (MDD), antidepressants can relieve only 22–50% of patients, and <33% of patients achieve clinical remission (1, 2). Identifying patients with early improvement potential before initiating medication can not only minimize unnecessary ineffective treatments but also optimize treatment compliance (3). Convergent evidence shows that early symptom improvement can robustly predict subsequent remission of MDD (4–7), while early non-responsiveness may indicate ultimate non-remission in response to antidepressants (8, 9). Therefore, the researchers suggest to advance the time window for evaluating early responses to 2 weeks (10, 11). In summary, exploring the prospective indicators of early response could optimize the “trial and error” method in current clinical routines (12).

Advances in resting-state functional magnetic resonance imaging (rs-fMRI) have substantially facilitated the identification of neuropathological features of depression (13–15). Emerging evidence has shown that spontaneous brain activity, which is reflected by the amplitude of low-frequency fluctuation (ALFF), is significantly different between MDD patients and healthy subjects (13, 16, 17). Compared with the measure of regional homogeneity, which describes the similarity of a given voxel time series to its nearest 26 neighbors (18), the ALFF was more suitable for measuring regional brain activity in the present study. Meanwhile, arterial spin labeling (ASL) imaging can reflect cerebral blood flow (CBF) *via* the non-invasive marking of arterial blood flow with an endogenous perfusion agent (19, 20). This method does not use a contrast tracer and is based upon the subtraction of two consecutively generated images. Accumulating evidence indicates the potential relevance of regional CBF and spontaneous activity (21, 22). However, it remains uncertain whether local hemodynamic changes and regional activities are altered simultaneously in MDD (23, 24).

Furthermore, some studies have suggested that pre-treatment functional features are distinct between responders and non-responders to antidepressant treatment (25). Meanwhile, several results have reported that antidepressants may impact cerebral perfusion in healthy subjects (26, 27), as well as the cortico-subcortical perfusion in remitted or treatment-resistant MDD patients (20, 28). However, predicting the antidepressant response using altered brain function in the pre-treatment phase has been challenging (29), and there is no study that has combined quantitative information from the CBF and ALFF to predict early antidepressants response. Therefore, the identification of multimodal predictors of early response may help bridge the gap between baseline imaging features and treatment efficacy (30).

We previously revealed that altered resting-state functions within the reward circuit and the default mode network (DMN) could allow for the identification of patients with unfavorable antidepressant responses (31, 32). In this study, we aimed to extend our previous research by predicting early treatment responsiveness [i.e., responsive depression (RD) or non-responsive depression (NRD)] using discriminative CBF and ALFF features and machine learning method. We also explored whether the abnormal CBF and ALFF in emotion-processing regions changed synchronously in MDD. We hypothesized that the alterations of CBF and ALFF are mainly located in frontal-limbic and subcortical regions, which are closely related to self-referential thought and maladaptive rumination in depression.

## Materials and Methods

### Participants

The participants were recruited from the Affiliated Zhongda Hospital, Southeast University, and the local community. The Southeast University Research Ethics Committee approved the research, and an informed consent form was signed by all subjects. All participants received MRI contradiction screening, demographic data, and clinical interviews. A consensus was reached by two trained experienced psychiatrists to diagnose MDD using the Structured Clinical Interview for DSM-IV Axis I Disorders (SCID-I/P), clinician version (33). The inclusion and exclusion criteria are the same as the items in our previous study (31) (more details are listed in the Supplementary Material). All participants are right-handed unequivocally and naturally. The MRI scans were processed before the administration of antidepressant treatment. During the 2-week follow-up period, five patients withdrew from the study. Finally, 80 MDD patients and 42 heathy controls (HCs) completed the procedures (2-week follow-up) and underwent the quality control (e.g., head motion and ghost intensity). According to the Hamilton Depression Scale (HAMD) score reduced by 50%, the MDD subjects were divided into the RD (*n* = 44) and NRD groups (*n* = 36) after 2 weeks of antidepressant treatment.

### Brain Image Acquisition

All participants were applied a 12-channel head coil to perform rs-fMRI and ASL imaging scans on a 3.0 Tesla scanner (Siemens Medical Systems, Erlangen, Germany). The subjects lie on their backs, and their heads are tightly secured with straps and foam pads to minimize potential head movement. All subjects were instructed to close their eyes, relax, and stay awake and not to consider any specific things during the scan. The details of MRI sequences are illustrated in the Supplementary Material.

### Functional Image Preprocessing Protocol

Resting-state functional images were preprocessed utilizing the Data Processing Assistant for Resting-State Function (DPARSF 2.3 Advanced edition) MRI toolkit (34), which combined procedures based on the Resting-State Functional MRI toolkit (REST, http://www.restfmri.net) (35), and statistical parametric mapping software package (SPM8, http://www.fil.ion.ucl.ac.uk/spm). Briefly, the slice timing, head motion checking, co-registering, spatial normalization, spatial smoothing, detrending, and nuisance signal (white matter, cerebrospinal fluid signal, rigid body-six-corrected head movement parameters) regression were conducted. After the above preprocessing, the averaged square root was obtained across 0.01–0.08 Hz at each voxel and the ALFF generated at the given voxel. Finally, for standardization procedure, ALFF was transformed to *Z* score by subtracting the global mean value and then divided by the standard deviation (36). The details are elaborated in the Supplementary Material.

The scrubbing was finally performed using the DPARSF. According to a previous study (37), if the volumes with framewise displacement (FD) larger than 0.5 mm with prior one and later two volumes will be deleted, then subjects with fewer than 4 min of remaining data (about 50% volumes) will be excluded.

To examine the potential confounder of head motion, we employed the mean FD (38) to control the contamination of head motion in group-level comparison. The FD measures the comparative head motion of each timepoint relative to the prior timepoint, by integrating six framewise head motion parameters (39).

The ASL image data were reconstructed and checked by two senior radiologists. T1 images were manually inspected for quality control, and artifacts were removed before preprocessing. The qualified PASL data was processed using the ASLtbx (40) and SPM12 (http://www.fil.ion.ucl.ac.uk/spm). The ALFF maps of each subject were transformed to *z*-maps with the REST software (http://www.restfmri.net) for further analysis. The results were corrected with the 3dClustSim program. For details, see Supplementary Material.

### Statistical Analyses

Continuous variables are presented as the mean ± standard deviation (SD). The analysis of covariance (ANCOVA), *post-hoc* test, independent two-sample *t*-test, and chi-square test (Statistical Package for the Social Sciences software, SPSS19.0, Chicago) were used to confirm significant differences in demographic data and HAMD scores among the RD, NRD, and HC groups. The imaging calculations were conducted in REST (35), and the results were corrected with the 3dClustSim program (https://afni.nimh.nih.gov/pub/dist/doc/program_help/ 3dClustSim.html). The group imaging comparison, correlation analyses, receiver operating characteristic (ROC), and support vector machine (SVM) analyses are detailed in the Supplementary Material. In this study, the “fingerprint” was employed to display three or more quantitative variable data in the form of a two-dimensional graph, which can visually illustrate the group data on an axis starting from the same point. Unless otherwise specified, the threshold of statistical significance was set as *P* < 0.05.

## Results

### Sample Characteristics

No significant differences in age, sex, or education levels were detected among the RD, NRD, and HC groups (all *P* > 0.05, Table 1), and no significant differences in baseline severity of depression (i.e., HAMD scores) were revealed between the RD and NRD groups (*t* = −0.477, *P* = 0.635). Furthermore, no significant differences in mean FD were exhibited among the three groups (*P* = 0.989).

**Table 1:** Demographic and clinical characteristics of all participants (mean ± SD).

**Group**	**RD (*n* = 44)**	**NRD (*n* = 36)**	**HC (*n* = 42)**	**Statistic value**	***P*** **value**	**Effect size**
Education (years)	9.27 ± 4.00	9.33 ± 4.28	11.02 ± 3.79	*F*_(2, 119)_ = 2.008	0.139^a^	*f* = 0.20
Age (years)	48.75 ± 13.31	47.64 ± 15.75	47.94 ± 17.62	*F*_(2, 119)_ = 0.112	0.894^a^	*f* = 0.03
Gender (F/M)	32/12	27/9	23/19	χ^2^ = 4.515, df = 2	0.105^b^	*w* = 0.19
HAMD	28.93 ± 7.35	29.67 ± 6.18	NA	*t* = −0.477, df = 78	0.635	*d* = 0.11
Handedness	Right	Right	Right	NA	NA	NA
Mean FD	0.107 ± 0.034	0.108 ± 0.029	0.108 ± 0.043	*F*_(2, 119)_ = 0.011	0.989	NA

*For comparison of demographics*:

a *P values were obtained using one-way ANOVA tests*;

b *P value for the gender distribution among three groups was obtained using chi-square test. P < 0.05 was considered significant. HC, healthy controls; NRD, non-responsive depression; RD, responsive depression; HAMD, Hamilton Depression Rating Scale; F/M, female/male; NA, not applicable; FD, framewise displacements. Parametric values are represented as the mean ± SD (standard deviation)*.

### The Common and Distinct Features of Regional CBF and ALFF Among the RD and NRD Groups

**Figure 1:**
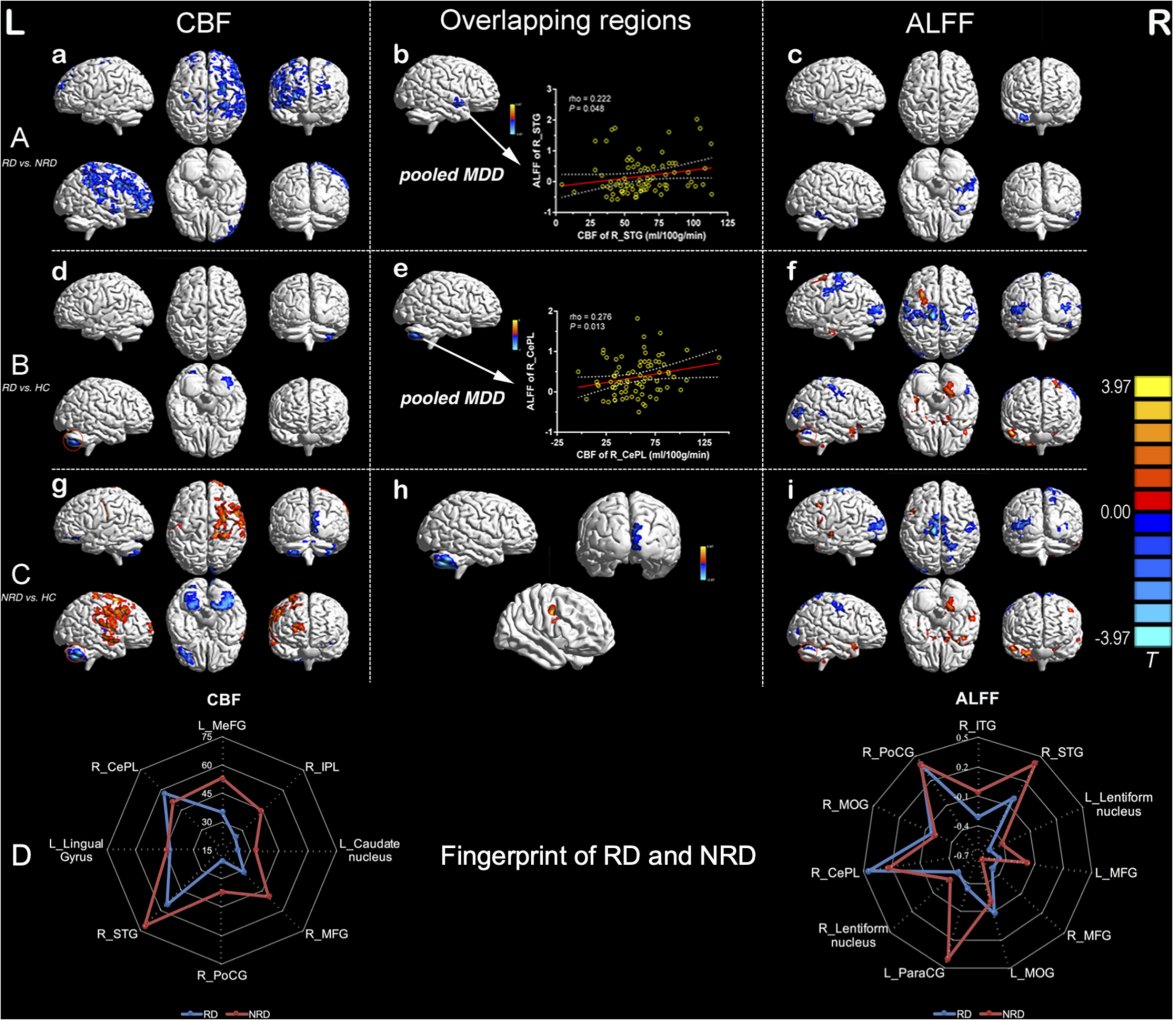
Inter-group regional differences in CBF and ALFF and positive correlations between CBF and ALFF values of overlapping regions (R_STG and R_CePL) in the pooled depression group. Row A: differences in regional CBF **(a)** and ALFF **(c)** between the RD and NRD and overlapping regions with significant differences between the CBF and ALFF modalities in pooled MDD **(b)**. Row B: differences in regional CBF **(d)** and ALFF **(f)** between RD and HC and overlapping regional alterations in CBF and ALFF in pooled MDD **(e)**. Row C: regional differences in CBF **(g)** and ALFF **(i)** between the NRD and HC and overlapping regions of the CBF and ALFF measures **(h)**; row D: the distinctive fingerprints of regional CBF and ALFF between the RD and NRD groups. The red circles in **(d)** and **(g)** indicate lower CBF and ALFF in R_CePL in both the RD and NRD groups, as compared with the HC group. The threshold was set at a corrected *P* < 0.05, and the *T*-score bar is present on the right side. CBF, cerebral blood flow; ALFF, amplitude of low-frequency fluctuation; L, left; R, right; HC, healthy controls; NRD, non-responsive depression; RD, responsive depression; STG, superior temporal gyrus; CePL, cerebellum posterior lobe.

As detailed in Figure 1, both the RD and NRD groups exhibited lower CBF in the left lingual gyrus and right posterior lobe of the cerebellum compared with the HC group (*P* < 0.05, 3dClustSim correction). The RD and NRD groups also showed lower ALFF in the bilateral sensorimotor cortex, bilateral middle occipital gyrus (MOG), and right posterior lobe of the cerebellum but presented higher ALFF in the bilateral middle frontal gyri (MFG) and lentiform nucleus when compared with the HC group (*P* < 0.05, 3dClustSim correction).

Distinct patterns of regional CBF and ALFF were also observed in the RD and NRD groups when compared with the HC group (Figure 1; Supplementary Tables S1, S2). Specifically, the RD group showed distinctly lower ALFF levels in the left postcentral gyrus (L_PoCG) and superior temporal gyrus (R_STG) but enhanced ALFF levels in the left precentral gyrus (L_PreCG) and left parahippocampal gyrus relative to the HC group.

In contrast to the RD group, the NRD group showed significantly lower CBF levels in the left superior frontal gyrus (L_SFG), right calcarine gyrus, and left cerebellum posterior lobe (L_CePL) but higher CBF in the right middle temporal gyrus (R_MTG), right middle/superior frontal gyrus, and R_ PoCG. As for the rs-fMRI modality, the NRD group exhibited lower ALFF levels in the right calcarine gyrus and right precuneus but greater ALFF levels in the left inferior frontal gyrus (L_IFG), R_PreCG, and R_STG compared with the HC group.

### The Distinct Fingerprints of Regional CBF and ALFF Between the RD and NRD Groups

To visually and comprehensively show the different patterns of CBF and ALFF between the RD and NRD groups, fingerprints were used to illustrate the regional changes in the ASL and BOLD modalities. Compared with the NRD group, the RD group showed distinct CBF patterns in the left frontal striatal area and right frontal cerebellar region and exhibited differential ALFF patterns in the left frontoparietal striatal area and right frontotemporal-striatal-cerebellar regions (Figure 1).

### Analysis of the Association Between the Imaging Measures and HAMD Scores

**Figure 2:**
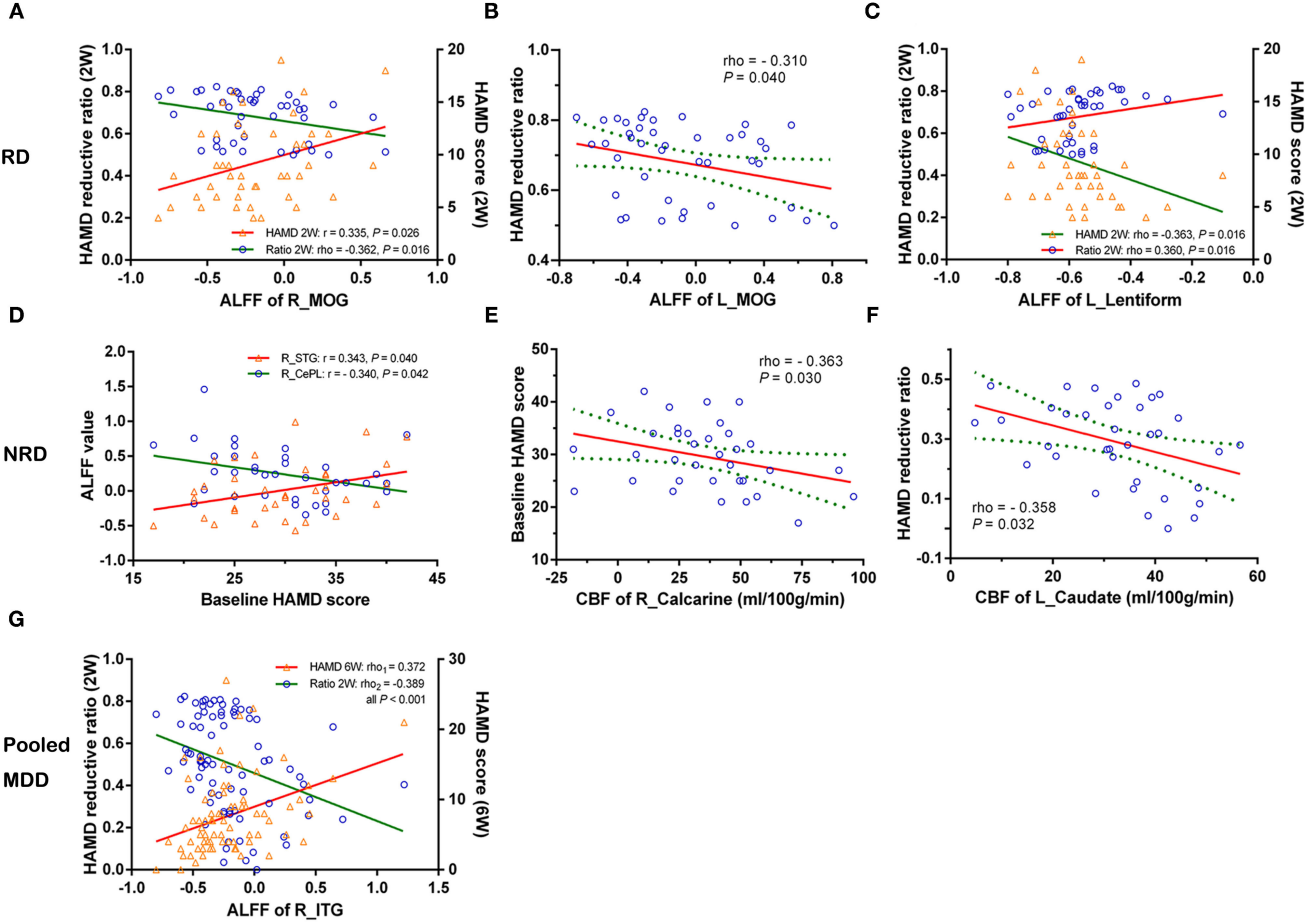
The correlations of changed CBF and ALFF and the severity/early improvement of depression in the RD, NRD, and pooled depression groups. Row RD: **(A)** decreased ALFF of the R_MOG was negatively related to the reduction of the HAMD score and positively correlated with the HAMD score (2W); **(B)** reduced ALFF of the L_MOG was negatively related to the reduction of the HAMD score; **(C)** increased ALFF of the L_lentiform nucleus was negatively related to the HAMD (2W) but positively correlated with the reduction of the HAMD score. Row NRD: **(D)** and **(E)** the baseline HAMD score was positively related to higher ALFF of the R_STG but negatively correlated with lower ALFF of the R_CePL and CBF of the R_calcarine gyrus, respectively. **(F)** The negative correlations between higher CBF of the L_caudate nucleus and the reduction of the HAMD score. Specifically, **(G)** represents that the ALFF of the R_ inferior temporal gyrus (R_ITG) was positively related to the HAMD (6W) but negatively correlated to the reduction of the HAMD score in the pooled MDD group (RD and NRD). CBF, cerebral blood flow; ALFF, amplitude of low-frequency fluctuation; L, left; R, right; RD, responsive depression; NRD, non-responsive depression; MDD, major depressive disorder; HAMD (2W/6W), Hamilton Depression Rating Scale score at week 2 or week 6; r/rho, Pearson's/Spearman's coefficient; MOG, middle occipital gyrus; STG, superior temporal gyrus; CePL, cerebellum posterior lobe; ITG, inferior temporal gyrus.

In the RD group, the lower ALFF in the right middle occipital gyrus (R_MOG) was negatively related to symptom improvement after 2 weeks of treatment (i.e., the HAMD reduction rate) (rho = −0.362, *P* = 0.016) and positively correlated with the HAMD score in week 2 (*r* = 0.335, *P* = 0.026) (Figure 2A). The lower ALFF in the L_MOG was negatively related to symptom improvements (rho = −0.310, *P* = 0.040) (Figure 2B). The higher ALFF of the L_lentiform nucleus was negatively related to the HAMD score in week 2 (rho = −0.363, *P* = 0.016) but positively related to symptom improvement (rho = 0.360, *P* = 0.016) (Figure 2C).

Additionally, in the NRD group, the baseline HAMD score was positively associated with the higher ALFF of the R_STG (*r* = 0.343, *P* = 0.040) but negatively correlated with the lower ALFF of the R_CePL (*r* = −0.340, *P* = 0.042) and the CBF of the R_calcarine gyrus (rho = −0.363, *P* = 0.030) (Figures 2D,E), respectively. A significant negative correlation was also found between the CBF of the L_caudate nucleus and symptom improvement (rho = −0.358, *P* = 0.032) (Figure 2F). Specifically, in the pooled MDD group (which included both the RD and NRD patients), the ALFF of the R_inferior temporal gyrus (R_ITG) was positively related to the HAMD score at week 6 (rho = 0.372, *P* < 0.001) but negatively correlated with the reduction of HAMD score (rho = −0.389, *P* < 0.001) (Figure 2G).

### The Predictive Performance of Regional CBF and ALFF Changes in Distinguishing the NRD From RD

**Figure 3:**
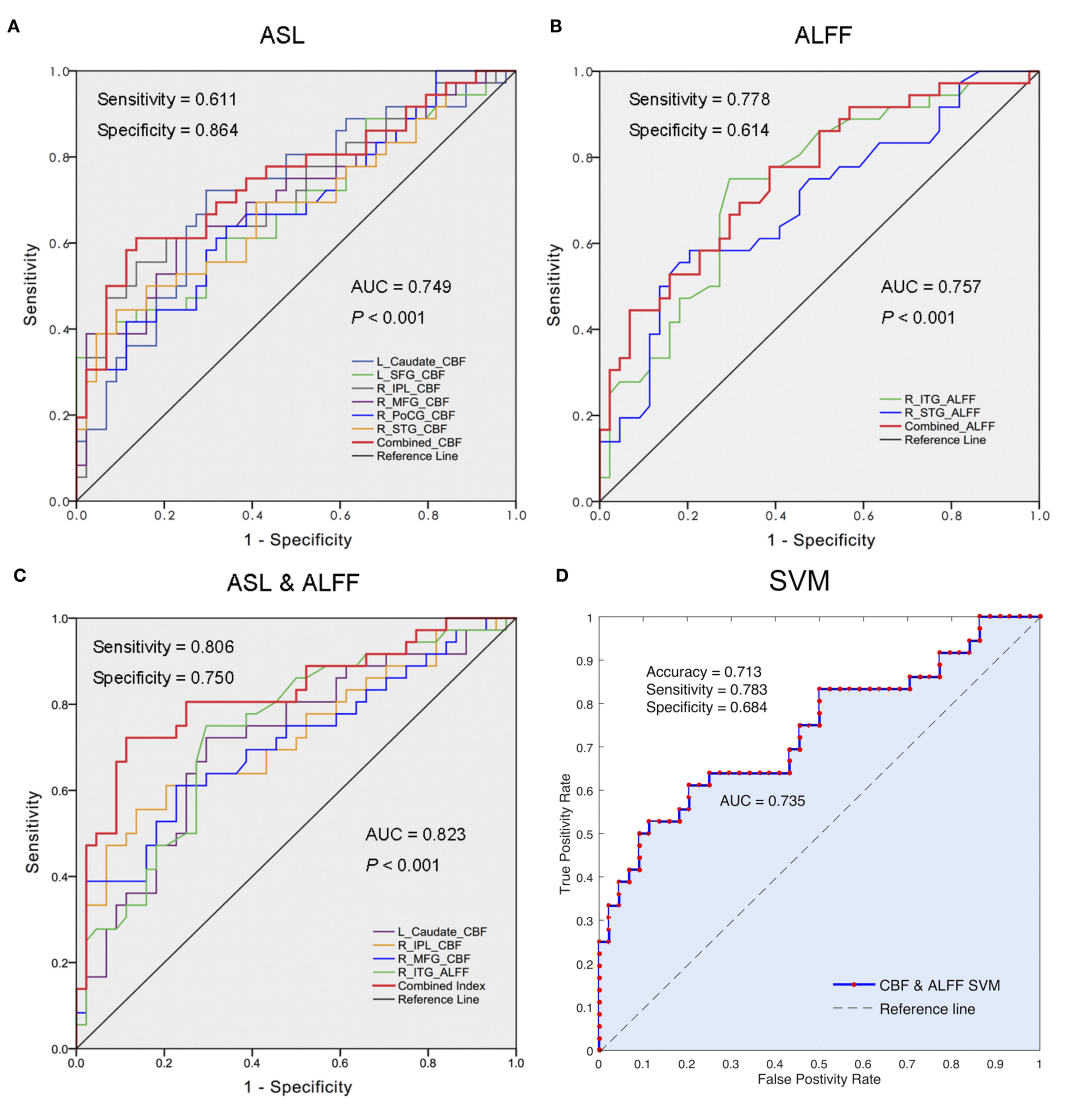
Differentiation analyses using regional CBF, ALFF, and integrated bi-modal parameters. **(A)** The performance of changed CBF values in differentiating the NRD from RD; **(B)** the performance of altered ALFF values in segregating the NRD from RD; **(C)** the optimal performance of the combination of changed CBF and ALFF values in distinguishing the NRD from RD; **(D)** the accuracy of the combined eight features (the CBF in L_caudate nucleus, R_SFG, R_IPL, R-MFG, R_STG, and R_PoCG, as well as the ALFF in R_STG and R_ITG) using 10-fold cross-validation SVM classifier. ROC, receiver operator characteristic; NRD, non-responsive depression; RD, responsive depression; AUC, area under the curve; CBF, cerebral blood flow; ALFF, amplitude of low-frequency fluctuation; L, left; R, right; SFG, superior frontal gyrus; IPL, inferior parietal lobule; MFG, middle frontal gyrus; PoCG, postcentral gyrus; STG, superior temporal gyrus; ITG, inferior temporal gyrus; SVM, support vector machine.

The ROC analyses revealed that the areas under the curve (AUCs) of the combined measures in each modality (i.e., CBF and ALFF) were 0.749 and 0.757, respectively (Figures 3A,B). When the measures of the two modalities (ASL and rs-fMRI) were integrated, they showed optimal performance for discriminating the NRD group from the RD group (AUC = 0.823, *P* < 0.001), with balanced specificity (89%) and sensitivity (72%) (Figure 3C). The ROC results of each regional measure are listed in Supplementary Table S3.

We further confirmed the classification power of the above eight features (CBF in the L_caudate nucleus, R_IPL, R_SFG, R-MFG, R_STG, and R_PoCG and ALFF in the R_ITG and R_STG) using a 10-fold cross-validation SVM classifier. The results revealed that the combination of eight features can discriminate the NRD patients with a moderate accuracy of 0.713 (AUC = 0.735, sensitivity = 0.783, specificity = 0.684) (Figure 3D).

## Discussion

In the present study, we primarily detected notable differences in widespread regions between the NRD and RD groups in the rs-fMRI and ASL modalities. Specifically, the fingerprint differences between the RD and NRD patients are mainly characterized by the CBF patterns in the left frontal striatal and right frontal cerebellar areas and by the ALFF features in the left frontoparietal striatal regions and the right frontotemporal striatal-cerebellar areas, respectively. The regional changes in CBF or ALFF in several regions were significantly correlated with depression severity or symptom improvement. Importantly, the ROC and SVM analyses using bi-modal (i.e., rs-fMRI and ASL) features further confirmed the optimal performance in differentiating NRD subjects, indicating the potential value of integrated imaging markers in predicting early antidepressant responses.

In the pooled MDD, the overlapping changes in the CBF and ALFF in the cerebellum when compared with the HC, which indicate that there were concomitant abnormalities in both blood perfusion and spontaneous activity in depression and further support the opinion that regional CBF is closely related to the intrinsic activity during emotion processing (22, 41). The cerebellum reciprocally connects with the limbic regions (42) and the DMN (43), which are responsible for emotion regulation and reappraisal (44). Imaging studies have substantiated that abnormal volume, brain activity, and interhemispheric coordination of the cerebellum are implicated in the pathophysiology and treatment of MDD (45, 46). Our results revealed that the lower ALFF in R_CePL was negatively related to the baseline severity of depression, further indicating that cerebellar dysfunction may be the neuropathological basis that affects the severity of depression.

Additionally, the present study found consistently lower CBF and ALFF in the occipital regions (left lingual gyrus, right calcarine gyrus, and bilateral MOG) and the sensorimotor regions in both the RD and NRD groups compared with the HC group. Those regions are considered essential components of bottom-up selective attention and visual perception in emotion regulation (47, 48). Aberrant activities in visual recognition circuits are deeply implicated in the dysregulation of facial emotion processing and pessimistic social interaction, mediating core symptoms of depression such as anhedonia and social oversensitivity (49, 50). Moreover, previous studies also confirmed reduced ALFF of the lingual gyrus and MOG in non-responsive (17) and treatment-resistant subjects (51). The present results extend the prior viewpoint that the dysfunction of facial emotion perception might partly constitute the neural substrates of MDD, which suggests a potential target for optimized medication.

Importantly, as an integral component of the DMN underlying the pathological rumination in depression, the R_STG showed lower CBF and ALFF in the RD group compared with the NRD group but did not differ significantly from the HC group. Evidence from functional imaging studies suggested that a higher CBF in the STG was critical to maintaining static DMN function in healthy subjects (52, 53). Furthermore, some fMRI studies have also found that the ALFF in the STG was reduced in treatment-naïve MDD (14) but was increased in the severe subtype of MDD with suicidality (54). Considering that an overactivated DMN substantially mediates maladaptive rumination and preferential attention bias to negative information in MDD (55), it would be reasonable to speculate that an abnormal hyperactivity of the STG is an essential neural substrate of severe depressive symptoms (e.g., intense suicidality) and inadequate treatment efficacy. Patients with favorable antidepressant efficacy may recruit compensatory downregulation of the STG to facilitate a positive reaction to antidepressant treatment (56).

The fingerprint patterns also revealed distinct CBF/ALFF changes between the RD and NRD groups, indicative of the involvement of spatially orchestrated neural ensembles in the neural mechanism of MDD. Those widespread regions are considered crucial mediators that can synergistically modulate the top-down process of emotion regulation and antidepressant efficacy in MDD (55, 57, 58). Importantly, the combined ROC analysis and 10-fold cross-validation SVM classification were further conducted. The results revealed stable performance with an optimal effect in discriminating the NRD from the RD group. In line with the evidence mentioned above (3, 58), the results further substantiated that multiple regions, rather than a specific single region, can mediate emotion regulation and identify patients with distinct neural features that may respond differently to antidepressant therapy.

As a critical nucleus of the corticostriatal circuit that is profoundly linked to emotion regulation, the caudate shows microstructural and functional aberrations in depressed patients (59) and in rats exposed to chronic mild stress (60). Furthermore, a recent study reported that reduced resting-state activity in the caudate could predict clinical remission in MDD after a single dose of antidepressants (61), suggesting that overactivation of the caudate could be selected as a potential target for antidepressants. Interestingly, the higher CBF of the L_caudate nucleus was also inversely correlated with symptom improvement in the NRD group. Our results further validated the prominent role of the caudate nucleus in antidepressant efficacy from the dimension of quantitative CBF. We also found that the ALFF of the R_ITG was positively related to the severity of depression in week 6 but was negatively correlated with early symptom improvement. The ITG is considered a pivotal region in the neuropathology of MDD, and recent rs-fMRI studies have detected abnormal network heterogeneity (62) and brain activation (63) of the R_ITG in MDD and dysfunction of the ITG in subthreshold depression (64). It is noteworthy that the ALFF value of the R-ITG in the RD group was significantly lower than that in the NRD group, indicating that the downregulation of spontaneous activity in the R-ITG might facilitate the improvement of depressive symptoms.

Some potential limitations should be considered when evaluating these results appropriately. First, the present study was designed without an MRI scan at the end of week 2, so whether these regional CBF/ALFF changes persist or could be reversed by antidepressant treatment needs to be further confirmed. Future studies using a longitudinal design are required to verify whether the group differences in the brain are causal or casual. Second, different antidepressants were prescribed in this study, so the prediction of treatment response to a specific antidepressant cannot be confirmed. Further studies investigating the predictors of early treatment response would benefit from the prescription of homogeneous types of antidepressants (e.g., SSRIs or SNRIs).

## Conclusion

In summary, the present study suggests that dysfunctions in distributed regions are orchestrated in the neuropathological processes of MDD, and the regional alterations of CBF and ALFF in the frontal striatal area may be promising markers for the prediction of short-term treatment outcomes. Meanwhile, the distinct alteration of the posterior lobe of the cerebellum [functionally connected to the DMN (65)] in the pooled MDD group may be a common trait in depression that can be considered a potential diagnostic marker of MDD. Although these findings need further validation before clinical application, they provide theoretical evidence for research domain criteria (RDoC) that adopt quantitative imaging features to predict efficacy during the early stage.

## Data Availability Statement

The raw data supporting the conclusions of this article will be made available by the authors, without undue reservation.

## Ethics Statement

The studies involving human participants were reviewed and approved by Southeast University Research Ethics Committee. The patients/participants provided their written informed consent to participate in this study.

## Author Contributions

YYu and ZH designed the study, had full access to the integrated data in the study, and took responsibility for the analysis of the data. WJ and YYi collected the clinical and MRI data. ZH and YZ performed the literature search, imaging data analyses, statistical analysis, and wrote the first draft of the manuscript. TL and XH helped to perform the machine learning analysis. All authors contributed to the article and approved the submitted version.

## Funding

This study was supported by Natural Science Foundation of Jiangsu Province (No. BK20201270 to ZH), National Natural Science Foundation of China (No. 81971277 to YYu and No.81801349 to YYi), Jiangsu Provincial Key Research and Development Program (No. BE2019748 to YYu), and State Scholarship Fund of China Scholarship Council (No. 201706090193 to ZH).

## Conflict of Interest

The authors declare that the research was conducted in the absence of any commercial or financial relationships that could be construed as a potential conflict of interest.

## Publisher's Note

All claims expressed in this article are solely those of the authors and do not necessarily represent those of their affiliated organizations, or those of the publisher, the editors and the reviewers. Any product that may be evaluated in this article, or claim that may be made by its manufacturer, is not guaranteed or endorsed by the publisher.
