# Supplementary material for: Distinct Features of Cerebral Blood Flow and Spontaneous Neural Activity as Integrated Predictors of Early Response to Antidepressants
Source: Front Psychiatry. 2022 Jan 18;12:788398. doi: 10.3389/fpsyt.2021.788398 (PMC8804095; doi:10.3389/fpsyt.2021.788398)
Supplement: Supplementary file 1 [file Data_Sheet_1.docx]

**Supplementary materials**

1. **Materials and Methods**

**1.1The inclusion and exclusion criteria**

Participants met the following inclusion criteria: (1) They met the DSM-IV criteria for major depression at the time of entry; (2) During the first depressive episode, the age of onset was over 18 years old; (3) 17 items Hamilton depression rating scale (HAMD) [1] were greater than 17; (4) No other major psychiatric illness, including substance abuse or dependence; (5) No primary neurological diseases, including dementia or stroke; (6) Absence of medical illness impairing cognition; (7) No history of receiving electroconvulsive therapy; (8) No claustrophobia or metal implants and other MRI contraindications; (9) T1-weighted image without gross structural abnormality, T2-weighted MRI without gross white matter Changes, such as infarction or other vascular lesions; (10) No psychotic symptoms (such ashallucination/bizarre delusions).Antidepressants are prescribed by psychiatrists based on the patient's condition, and researchers do not intervene in the clinical decision-making of treatment strategies.The treatment inMDD group was as follows: 48 patients received selective serotonin reuptake inhibitor (SSRIs), 26 patients with serotonin-norepinephrine reuptake inhibitor (SNRIs) and 6 patients received algorithm of antidepressants combinations (SSRIs, SNRIs or mirtazapine).All patients were evaluated for HAMD at week 0, week 2.After removing head movement or poor image quality (i.e., ghosting intensity), 80 MDD patients and 42 healthy controls (HC) completed the procedures in this study.

**1.2 Brain image acquisition**

High-resolution 3-dimensional T1-weighted scans were recorded as magnetization prepared rapid gradient echo (MPRAGE) sequence: repetition time (TR) = 1900ms, echo time (TE) = 2.48ms; flip angle (FA) = 9°; field of view (FOV) = 250 × 250mm^2^; acquisition matrix = 256 × 256; thickness = 1.0mm, gap = 0;176 slices; time = 4 minutes 18 seconds. The ASL MRI was performed using the Siemens product PASL PICORE Q2T sequence: TR = 4000ms; TE = 12ms; TI (inversion time)_1_ = 600ms; TI_2_ = 1600ms; FOV = 220 × 220mm^2^;acquisition matrix = 64 × 64;FA = 90°; 27 axial slices;gap = 1mm; thickness = 4mm; time = 7 minutes 14 seconds. The acquisition parameters of rs-fMRI were as follows: TR = 2000 ms; TE = 25 ms; FA = 90°;field of view = 240 × 240 mm^2^; matrix = 64 × 64; thickness = 3.0 mm; gap = 0 mm; 240 volumes,36 axial slices, and 3.75 × 3.75 mm^2^ in-plane resolution parallel to the anterior commissure-posterior commissure line, scan time = 8 minutes.

**1.3Functional image preprocessingprotocol**

The first 10 time-points were removedin order to ensure stable-state longitudinal magnetization and adaptation to inherent scanner noise. The remaining 230rs-fMRI images were sequentiallyperformed according to following steps: (1) slice timed with the 35th slice as reference slice; corrected for temporal differences and head motion (subjectswith head motion of more than 1.5 mm of maximum displacement in any direction (x, y, or z) or 1.5 degrees of angular motion were excluded); (2)co-registered T1 to functional image and then reorientedthem; (3) spatial normalization: segment the T1-weighted anatomical image into white matter, gray matter, and cerebrospinal fluid, and then used unified the transformation parameters estimated by the segmentation algorithm were normalized to the space of the MontrealNeurology Institute (MNI)[2]. Applied the above transformation parameters to the functional image, and then resample the functional image with 3 mm isotropic voxels; (4) spatial smoothingundertaken with a 6 mm full-width at half-maximum(FWHM)isotropic Gaussian kernel; (5) the linear trend within each voxel’s time series removed; (6) nuisance signals (white matter, cerebrospinal fluid signal, rigid body-6 corrected head-movement parameters) and spike regressorswere regressed; (7) temporal bandpass (0.01-0.08Hz) to eliminateundesiredlow-frequency drift andfiltered high-frequency noise.

To avoid detecting spurious motion artifacts, labeling and control images were processed independently[3, 4]. High pass filtering (cut-off=0.5) was used for temporal PASL images. Then thePASL images were co-registered with the T1 imagesand the 6 mm FWHM kernel was employed for spatial smoothing for subsequent image subtraction.After outlier removing, the PASL images were masked to the MNI template in SPM12andcleanextra-brain voxels.

Then, the square root of the power spectrum was computed and then averaged across a predefined frequency interval.For ALFF analysis, we used Fast Fourier Transform to convert the time series of a given voxel to the frequency domain without bandpass filtering[5]. Then, wecalculatedthe square root of the power spectrum, and taken the average value over a predefined frequency interval.

**1.4 Statistical analysesdd**

The Cohen’s d values, which reflect the effect size of statistical difference between groups, was calculated by G*Power software 3.1 [6]. For comparisons of imaging data, the differences among the RD, NRD, and HC groups were analyzedby ANCOVA within groupmaskofgray matter,andage, gender and education level wereusedas covariates to control the confounding factors. Subsequently, the regions with significant differences in ANCOVA were saved as masks, which were used to calculate the group differences of CBF and ALFF through the*post hoc* two-sample t-tests. The above imaging calculations were conducted in REST and the results were corrected with the3dClustSim program(*P*<0.05,cluster size≥ 35 voxels, see Analysis of Functional NeuroImages(AFNI),https://afni.nimh.nih.gov/pub/dist/doc/program_help/3dClustSim.html) within the unilateral hemisphere of symmetric template. And then, the relationships between the changed CBF/ALFF and clinical characteristic were determined by bivariate Pearson’s/Spearman’s analysis.According to the numbers of variates include in analysis, *P*< 0.001 waschoseas threshold of significance.

Finally, the predictive performance of the altered imaging parameterswerecalculated,including the sensitivity, specificity and area under the receiver operating characteristic (ROC) curves (AUC: 0.9-1.0 =excellent; 0.8-0.9= well; 0.7-0.8=fair; 0.6-0.7=poor; 0.5-0.6=fail)[7]. Optimal cut-off between sensitivity and specificity were determinedby maximizing the Youden’s index J (J=sensitivity+specificity-1)[8].As we conducted in previous study, the binary logistic regression analysis was applied to obtain the combined distinguishing effect of the altered CBF/ALFF.

Furthermore, to validate the classificationpower of the combinedCBF and ALFF measures, the machine learning method (SVM classifier, cost=1,gamma=0.125) was employed to distinguishthe RD and NRDin the LIBSVMlibrary[9]inMatlab 8.6(Matlab R2015b, The MathWorks, Inc., Natick, Mass., https://www.mathworks.com/products/new_products/r2015b). The 10-fold cross-validation (CV) method was applied to determine the robust performance of the proposed model which can avoid the issue of over-fitting [10]. The entire dataset was randomly divided into 10 subsets. Any 9/10 subsets were selected as training data, and the remaining 1 subset was usedas testing data.Generally, the 10-fold CV was repeated 10 times to control the bias introduced by the random parcellation of the dataset. Finally, thesensitivity, specificity and accuracywere generated by averaging the classification results.

1. **Results**

**2.1 The overlapping regions between the alteredCBF andALFF**

LowerCBF and ALFF in the R_STG werefound in the RD as compared with the NRD. Moreover, as compared with the HC group, both RD and NRD groups showed lowerCBF and ALFF in R_CePL.

In the pooled MDD group(RD plus NRD), the CBF value of the R_ STG and the R_CePL were positively correlated with the corresponding ALFF value, respectively (r = 0.222, *P* = 0.048 (uncorrected) and rho = 0.276, *P* = 0.013 (Bonferroni corrected))(**Figure 1b,1e**).

In the NRD group,lowerCBF and ALFF in right calcarine gyrus and right middle frontal gyrus were found as compared to the HCgroup(*P*< 0.05, 3dClustSim correction)(**Table S1& S2**).

**2.2** **The ROC results of each regional measures**

The brain regions with significant difference in the CBF or ALFF between the RD and NRD were selected as the classification index for further ROC analyses. Specifically, the results demonstrated that the changed CBF in L_caudate nucleus (AUC = 0.715, P = 0.001), right inferior parietal lobule (R_IPL) (AUC = 0.721, P = 0.001), right middle frontal gyrus (R_MFG) (AUC = 0.707, *P* = 0.001) and the changed ALFF in R_ITG (AUC = 0.736, *P*< 0.001) reach the threshold (AUC > 0.700)of fair performance on discriminating the NRD from RD patients (**Table S3**).

**References**

[1] Hamilton M. A rating scale for depression. Journal of neurology, neurosurgery, and psychiatry. 1960;23:56-62.

[2] Ashburner J, Friston KJ. Unified segmentation. NeuroImage. 2005;26:839-51.

[3] Wang Z, Aguirre GK, Rao H, Wang J, Fernandez-Seara MA, Childress AR, et al. Empirical optimization of ASL data analysis using an ASL data processing toolbox: ASLtbx. Magn Reson Imaging. 2008;26:261-9.

[4] Wang Z. Improving cerebral blood flow quantification for arterial spin labeled perfusion MRI by removing residual motion artifacts and global signal fluctuations. Magn Reson Imaging. 2012;30:1409-15.

[5] Zou QH, Zhu CZ, Yang Y, Zuo XN, Long XY, Cao QJ, et al. An improved approach to detection of amplitude of low-frequency fluctuation (ALFF) for resting-state fMRI: fractional ALFF. J Neurosci Methods. 2008;172:137-41.

[6] Faul F, Erdfelder E, Lang AG, Buchner A. G*Power 3: a flexible statistical power analysis program for the social, behavioral, and biomedical sciences. Behav Res Methods. 2007;39:175-91.

[7] Beck JR, Shultz EK. The use of relative operating characteristic (ROC) curves in test performance evaluation. Arch Pathol Lab Med. 1986;110:13-20.

[8] Galen RS. Application of the predictive value model in the analysis of test effectiveness. Clin Lab Med. 1982;2:685-99.

[9] Chang CC, Lin CJ. LIBSVM: A library for support vector machines. ACM Transactions on Intelligent Systems and Technology. 2011;2:1-27.

[10] Plonski P, Gradkowski W, Altarelli I, Monzalvo K, van Ermingen-Marbach M, Grande M, et al. Multi-parameter machine learning approach to the neuroanatomical basis of developmental dyslexia. Human brain mapping. 2017;38:900-8.

**Table S1. The differences of CBF in the ASL modality among three groups**.

| **Brain regions** | **BA** | **Voxel number** | **Coordinates MNI** | | | **T-score** |
| --- | --- | --- | --- | --- | --- | --- |
|  |  |  | **X** | **Y** | **Z** |  |
| ***RD vs. NRD*** |  |  |  |  |  |  |
| L_SuperiorFrontal Gyrus | 9 | 70 | -18 | 48 | 36 | -3.621 |
| R_MiddleFrontal Gyrus | 6 | 474 | 30 | 3 | 63 | -4.039 |
| L_MedialFrontal Gyrus | 6 | 209 | 0 | -45 | 54 | -3.927 |
| R_PostcentralGyrus | 4 | 61 | 30 | -30 | 69 | -3.187 |
| R_SuperiorTemporal Gyrus | 21 | 45 | 57 | 9 | -12 | -3.501 |
| R_InferiorParietal Lobule | 40 | 214 | 69 | -18 | 15 | -4.215 |
| L_CaudateNucleus | / | 43 | -15 | 15 | 18 | -3.216 |
| ***RD vs. HC*** |  |  |  |  |  |  |
| L_Lingual Gyrus | 17 | 50 | -24 | -87 | -12 | -3.232 |
| R_CerebellumPosterior Lobe | / | 52 | 33 | -69 | -42 | -2.971 |
| ***NRD vs. HC*** |  |  |  |  |  |  |
| L_SuperiorFrontal Gyrus | 11 | 39 | -39 | 33 | -18 | -3.496 |
| R_CalcarineGyrus | 17 | 91 | 12 | -96 | 12 | -4.001 |
| L_LingualGyrus | 17 | 52 | -15 | -90 | -3 | -3.470 |
| L_CerebellumPosterior Lobe | / | 67 | -15 | -48 | -42 | -3.849 |
| R_CerebellumPosterior Lobe | / | 245 | 42 | -69 | -42 | -4.099 |
| R_MiddleTemporal Gyrus | 21 | 65 | 57 | -3 | -15 | 3.056 |
| R_Middle/Superior Frontal Gyrus | 6/8 | 169 | 30 | 6 | 63 | 4.188 |
| R_PostcentralGyrus | 4 | 36 | 33 | -30 | 72 | 3.175 |

**Abbreviations:** RD, responsive depression; NRD, non-responsivedepression;HC, healthy controls; ASL,arterial spin labeling; CBF,cerebral blood flow; L, left; R, right; BA, Brodmann area; MNI,Montreal neurological institute. The threshold has been set at a corrected *P*<0.05 (3dClustSim correction).

**Table S2. The differences of ALFF in thers-fMRI modality among three groups.**

| **Brain regions** | **BA** | **Voxel number** | **Coordinates MNI** | | | **T-score** |
| --- | --- | --- | --- | --- | --- | --- |
|  |  |  | **X** | **Y** | **Z** |  |
| ***RD vs. NRD*** |  |  |  |  |  |  |
| R_SuperiorTemporal Gyrus | 21 | 40 | 36 | 18 | -33 | -3.361 |
| R_ Inferior Temporal Gyrus | 20 | 60 | 39 | -27 | -30 | -3.676 |
| ***RD vs. HC*** |  |  |  |  |  |  |
| L_ Postcentral Gyrus | 4 | 195 | -36 | -36 | 48 | -4.190 |
| R_ Postcentral Gyrus | 4 | 92 | 57 | -18 | 42 | -3.615 |
| R_ Superior Temporal Gyrus | 22 | 62 | 54 | -33 | 0 | -3.713 |
| L_Paracentral Lobule | 6 | 59 | -6 | -24 | 78 | -4.450 |
| L_MiddleOccipital Gyrus | 19 | 173 | -36 | -87 | 9 | -3.742 |
| R_ Middle Occipital Gyrus | 19 | 56 | 39 | -90 | 15 | -3.291 |
| R_ CerebellumPosterior Lobe | / | 52 | 33 | -69 | -42 | -2.971 |
| L_ Middle Frontal Gyrus | 6 | 47 | -24 | 3 | 66 | 3.594 |
| R_ Middle Frontal Gyrus | 9 | 55 | 39 | 0 | 39 | 3.784 |
| L_Lentiform Nucleus | / | 90 | -18 | 6 | -6 | 3.922 |
| R_Lentiform Nucleus | / | 108 | 24 | -3 | -6 | 4.722 |
| L_Parahippocampal Gyrus | 20 | 247 | -42 | -15 | -24 | 4.206 |
| L_PrecentralGyrus | 6 | 101 | -48 | -3 | 18 | 4.067 |
| ***NRD vs. HC*** |  |  |  |  |  |  |
| R_ Postcentral Gyrus | 4 | 83 | 54 | -12 | 48 | -3.033 |
| L_Paracentral Lobule | 6 | 260 | -9 | -21 | 78 | -4.506 |
| R_CalcarineGyrus | 17 | 36 | 15 | -75 | 15 | -3.029 |
| R_Precuneus | 7 | 42 | 18 | -69 | 51 | -3.561 |
| L_ Middle Occipital Gyrus | 19 | 215 | -36 | -84 | 9 | -3.807 |
| R_ Middle Occipital Gyrus | 19 | 48 | 36 | -81 | 3 | -3.278 |
| R_CerebellumPosterior Lobe | / | 41 | 33 | -63 | -33 | -3.123 |
| L_ Middle Frontal Gyrus | 9 | 42 | -21 | 24 | 36 | 3.857 |
| R_ Middle Frontal Gyrus | 9 | 54 | 30 | 21 | 36 | 4.000 |
| L_InferiorFrontal Gyrus | 6 | 163 | -45 | -3 | 24 | 3.809 |
| R_ Precentral Gyrus | 6 | 40 | 36 | -3 | 36 | 4.375 |
| R_ Superior Temporal Gyrus | 38 | 69 | 33 | 15 | -36 | 3.955 |
| L_Lentiform Nucleus | / | 280 | -18 | 6 | -9 | 4.479 |
| R_Lentiform Nucleus | / | 102 | 24 | -3 | -12 | 4.093 |

**Abbreviations:** RD, responsive depression; NRD, non-responsivedepression;HC, healthy controls; rs-fMRI, resting-state functional magnetic resonance imaging; ALFF,amplitude of low frequency fluctuation; L, left; R, right; BA, Brodmann area; MNI,Montreal neurological institute. The threshold has been set at a corrected *P*<0.05 (3dClustSim correction).

**Table S3.Theperformance of integrated indexes in differentiating the NRD from RD.**

| **Parameter** | **AUC** | ***P*** | **95% CI** | **Sensitivity** | **Specificity** | **Cut-point** |
| --- | --- | --- | --- | --- | --- | --- |
| **L_Caudate_CBF** | 0.715 | 0.001 | 0.60-0.83 | 0.72 | 0.71 | 28.03 |
| **R_IPL_CBF** | 0.721 | 0.001 | 0.61-0.84 | 0.56 | 0.86 | 42.75 |
| **R_MFG_CBF** | 0.707 | 0.001 | 0.59-0.82 | 0.61 | 0.77 | 41.41 |
| **R_ITG_ALFF** | 0.736 | 0.000 | 0.63-0.85 | 0.75 | 0.71 | -0.26 |
| **Combined index** | 0.823 | 0.000 | 0.73-0.92 | 0.72 | 0.89 | 0.49 |

**Abbreviations:** ROC, receiver operator characteristic; NRD, non-responsivedepression; RD, responsive depression; AUC, area under curve; 95% CI, 95% confidence interval; CBF, cerebral blood flow; ALFF, amplitude of low frequency fluctuation; L, left; R, right; IPL, inferior parietal lobule; MFG, middle frontal gyrus; ITG, inferior temporal gyrus. The AUC of combined index is acquired from the integrated predictive effects of above bimodal imaging parameters.
